# An High-Throughput *In Vivo* Screening System to Select H3K4-Specific Histone Demethylase Inhibitors

**DOI:** 10.1371/journal.pone.0086002

**Published:** 2014-01-29

**Authors:** Cecilia Mannironi, Marco Proietto, Francesca Bufalieri, Enrico Cundari, Angela Alagia, Svetlana Danovska, Teresa Rinaldi, Valeria Famiglini, Antonio Coluccia, Giuseppe La Regina, Romano Silvestri, Rodolfo Negri

**Affiliations:** 1 Istituto di Biologia e Patologia Molecolari Consiglio Nazionale delle Ricerche, Rome, Italy; 2 Istituto Pasteur Fondazione Cenci Bolognetti, Dipartimento di Biologia e Biotecnologie “C. Darwin”, Sapienza Università di Roma, Rome, Italy; 3 Dipartimento di Chimica e Tecnologie del Farmaco, Sapienza Università di Roma, Rome, Italy; Tulane University Health Sciences Center, United States of America

## Abstract

**Background:**

Histone demethylases (HDMs) have a prominent role in epigenetic regulation and are emerging as potential therapeutic cancer targets. The search for small molecules able to inhibit HDMs *in vivo* is very active but at the present few compounds were found to be specific for defined classes of these enzymes.

**Methodology/Principal Findings:**

In order to discover inhibitors specific for H3K4 histone demethylation we set up a screening system which tests the effects of candidate small molecule inhibitors on a *S.cerevisiae* strain which requires Jhd2 demethylase activity to efficiently grow in the presence of rapamycin. In order to validate the system we screened a library of 45 structurally different compounds designed as competitive inhibitors of α -ketoglutarate (α-KG) cofactor of the enzyme, and found that one of them inhibited Jhd2 activity *in vitro* and *in vivo*. The same compound effectively inhibits human Jumonji AT-Rich Interactive Domain (*JARID) 1B* and *1D in vitro* and increases H3K4 tri-methylation in HeLa cell nuclear extracts (NEs). When added *in vivo* to HeLa cells, the compound leads to an increase of tri-methyl-H3K4 (H3K4me3) but does not affect H3K9 tri-methylation. We describe the cytostatic and toxic effects of the compound on HeLa cells at concentrations compatible with its inhibitory activity.

**Conclusions/Significance:**

Our screening system is proved to be very useful in testing putative H3K4-specific HDM inhibitors for the capacity of acting *in vivo* without significantly altering the activity of other important 2-oxoglutarate oxygenases.

## Introduction

Chromatin structure governs several aspects of cell metabolism. Histone N-terminal tails are subjected to several covalent modifications which form a sophisticated combinatory code which is read and interpreted by a plethora of regulatory protein complexes [1], [2]. Among the various modifications, Lysine (K) methylation is particularly interesting, due to its widespread roles in transcriptional regulation, DNA repair and epigenetic inheritance [3]. In *S.cerevisiae*, three lysine methyl transferases, Set1, Set2 and Dot1, catalyse histone mono-, di- or tri-methylation at K4, K36 and K79, respectively. These epigenetic marks, which are absolutely conserved among eukaryotes, have been associated with actively transcribed loci [4], although their roles in controlling transcription efficiency may be distinct and strongly context-dependent [5].

In particular, H3K4 tri-methylation is enriched at the promoters and 5′ portions of actively transcribed open reading frames in both yeast and higher eukaryotes [6] and seems to play multiple, variable and sometime conflicting roles in transcription [5], [7]–[10].

In higher eukaryotes methylation of H3K9 and K27 residues is strictly coupled to transcriptional repression and silencing [2], although these modifications are not observed in *S.cerevisiae* [1].

For many years, histone lysine methylation has been considered irreversible and persisting through cell division. Recently, two families of HDMs have been identified in eukaryotes: the Lysine Specific histone Demethylase 1 (LSD1) family and the Jmjc-domain-containing family [11]–[14]. The LSD1 HDMs are monoamine oxidases that can demethylate mono- and di-methylated H3K4 and H3K9 and require flavin adenine dinucleotide (FAD) for their function [13]–[15]. On the other end, Jumonji C domain-containing HDMs (JHDMs), 5 members in *S.cerevisiae* and at least 27 members in *H.sapiens*, are Fe^2+^ and α-KG-dependent hydroxylases, and their reported substrate residues include H3K4, H3K9, H3K27, and H3K36 at all methylation states [11], [12]. The JHDM Jhd2 (encoded by YJR119c and also called Lysine specific Demethylase 5, KDM5) was purified from budding yeast and was shown to specifically remove H3K4 di- and tri-methylation [16]–[18]. JHDMs are potential therapeutic cancer targets [19] and among them, those capable to demethylate specifically H3K4 (*JARID1A-1D*, [20] look particularly interesting. Indeed, at least one of these enzymes is strictly associated with human cancer: KDM5A (also known as *RBP2* and *JARID1A*) is over-expressed in gastric cancer, and its inhibition triggers cellular senescence of gastric cancer cells [21]. In acute myeloid leukemia (AML), KDM5A has been shown to form a fusion protein with a nucleoporin 98 gene (NUP98), and over-expression of this fusion protein alone is sufficient to induce AML in murine models. Furthermore, genetic ablation of KDM5A decreases tumor formation and prolongs survival in pRB-defective mice [22]. Very recently, KDM5A was found to be a critical epigenetic factor for the development of drug resistance in lung and breast cancer cells [23], [24]. Other *JARID* HDMs may be involved in cancerogenesis. *JARID1B* is up-regulated in 90% of human breast cancers and recently it has been shown to promote breast tumor cell cycle progression through epigenetic repression of microRNA let-7e [25]. Both *JARID1A* and *JARID1B* appear to contribute to retinoblastoma-mediated gene silencing during cellular senescence [26]. The search of *in vivo* inhibitors of *JARID* enzymatic activity is therefore very active, although only one of the HDM inhibitors which were found so far was shown to specifically inhibit H3K4 modification *in vivo* and *in vitro* [27], while all the others [28]–[31] seem to have more general and pleiotropic effects. In order to screen for specific H3K4 demethylase inhibitors, we developed an experimental system based on *S.cerevisiae*, taking advantage of the fact that this yeast contains only one H3K4-specific JHDM, i.e. Jhd2. The stringent requirement on Jhd2 demethylase activity of a particular strain to grow in the presence of rapamycin allowed to detect the possible inhibitory activity of 45 compounds, selected by a computer-driven drug design approach, by determining their cytostatic effects on yeast cells.

## Materials and Methods

### Yeast strains and plasmids

All *S.cerevisiae* strains and plasmids used in this work are reported in Table 1 and Table 2, respectively.

**Table 1:** Yeast strains.

Yeast Strain	Genotype	Reference
*BY4741*	*MATα; his3Δ1; leu2Δ0; lys2Δ0; ura3Δ0*	Euroscarf
*Δnot4*	*BY4741; MATα; his3Δ1; leu2Δ0; lys2Δ0; ura3Δ0; YER068w::kanMX4*	Euroscarf
*Δjhd2*	*BY4741; MATα; his3Δ1; leu2Δ0; lys2Δ0; ura3Δ0; YJR119c::kanMX4*	Euroscarf
*SDBY1066*	*BY4741: MATα his3Δ leu2Δ0 LYS2 met15Δ0 ura3Δ0 not4Δ::KanMX jhd2Δ::HygMX*	Ref.[36]
SDBY1066 with pDPM2	*BY4741 MATα; his3Δ; leu2Δ0;LYS met15Δ0 ura3Δ0; not4Δ::KanMX; jhd2Δ::HygMX with pDPM2*	Ref.[36]
*MBY1282* with pDPM4	*MATα; his3Δ2; ade2::hisG leu2Δ0; ura3Δ0; met15Δ0; trp1Δ63; Ty1his3Al-236; Ty1ade2Al-515; SET1-N-3XMYC; with pDPM4*	Ref.[36]
*YCVS3* with pDPM4	*MATα; ade2-1; ura3-1; his3-11; trp1-1; leu2_3,112; can1-100; set1::ura3*	Ref. [52] (with pDPM4 - this study)

**Table 2:** Plasmids.

Plasmid	Inserted Gene	Promoter	Vector	Source
pDPM2	JHD2-FLAG	JHD2p	pRS415	Ref.[36]
pDPM4	JHD2-FLAG	*PYK1p*	pDPM1/PYK1p	Ref.[36]

### Design and synthesis of candidate inhibitors

Molecular modeling studies were performed on a MacPro dual 2.66 GHz Xeon running Ubuntu 12. The crystal structures were downloaded from the PDB (http://www.rcsb.org/). Hydrogen atoms were added to the protein, using Molecular Operating Environment (MOE) 2007.09. (http://www.chemcomp.com/). Ligand structures were built with MOE and minimized using the MMFF94x force field. The docking simulations were performed using PLANTS [32] and Autodock [33]. The *JARID*, Jhd2 and JMJD2 JmjC domain sequences were retrieved by the Uniprot database (http://www.uniprot.org/). Structure alignment and homology modeling were performed by MOE. The best score models were then selected for docking studies.

### Screening of Jhd2 inhibitors in S.cerevisiae

Exponentially growing cells from the double deletion strain *SDBY1066* (*Δjhd2-Δnot4*) transformed with pDPM2 were inoculated at a cell density corresponding to 0.2 OD^600^ in a 96 microtiter plate wells containing 200 µl of 2% Bacto-peptone 1% Bacto-yeast extract, 3% Glucose (YPD) plus 50 nM rapamycin. Each test compound of the library was dissolved in dimethyl sulfoxide (DMSO) and added at 5 or 15 µM final concentration and OD^600^ was monitored at 24 h and 48 h after incubation at 30°C. The following controls were added: untreated pDPM2-transformed *SDBY1066* strain; wild type *BY4741* strain with and without 50 nM rapamycin and DMSO at the same percentage as for the inhibitor dilutions (0.5% or 1.5%) and wild type strain treated with each inhibitor (15 µM) in the presence of rapamycin. Alternatively, cell growth was monitored in 50 ml liquid cultures of YPD containing the indicated concentrations of rapamycin, DMSO or a candidate inhibitor, inoculated with exponentially growing cells at a cell density corresponding to 0.2 OD^600^ with constant shaking. Cell growth was monitored for 8 h at 30°C.

### Preparation of S.cerevisiae Cell Free Extract (CFE)

Cells from pDPM2-transformed *SDBY1066* strain or from pDM4-trasformed *YCVS3* strain were grown in YPD at a cell density corresponding to 0.8 OD^600^ and pelleted. The cells were subsequently washed two times and resuspended in 0.5 ml of 50 mM Hepes (pH 8); 50 mM KCl; 1 mM EDTA, 10% glycerol and protease inhibitors (complete EDTA-free Protease Inhibitor Cocktail, Roche). Samples were added with equal volumes of glass beads (Sigma G8772, diameter: 425–600 µm) and vortexed 8 times for 2 min at 4°C with 1 min intervals. Lysates were subsequently recovered and cleared by centrifugation at 3000 g.

### Preparation of HeLa NE

NE from HeLa cells were prepared using the Nuclear Extract Kit (Active Motif) according to the standard protocol.

### Testing demethylase activity on S.cerevisiae or HeLa cells NE

The indicated volumes of NE were added to 10 µl of reaction containing 5 µg of purified calf thymus histones (from Sigma Aldrich) in 50 mM Hepes (pH 8), 1 mM α-KG, 0.1 mM Fe_2_SO_4_, 2 mM ascorbate containing protease inhibitors (Complete EDTA-free Protease Inhibitor Cocktail, Roche). The candidate inhibitor DMSO dissolved was tested at different concentrations as indicated (DMSO 2.5% of reaction volume). 5-deoxy-5-methylthioadenosine (MTA) final concentration was 100 µM. Reactions were kept 5 h and 3 h at 37°C for yeast and HeLa cell extracts, respectively. Reactions were stopped by 2× Laemmli loading buffer addition and directly loaded on gels for western blot analysis.

### Testing compound 3195 inhibitor on purified JARID enzymes

Purified human recombinant *JARID*1B/Plu-1 (BPS Bioscience) (240 ng), or *JARID*1D (Abnova) (240 ng) or KDM6B (Sigma-Aldrich) (80 ng) were added to 15 µl of a reaction mixture containing 5 µg of purified calf thymus histones (Sigma Aldrich) in 50 mM Tris HCl pH 7.5); 1 mM α-KG; 0.1 mM (NH_4_)_2_Fe(SO_4_)_2_; 2 mM ascorbate; protease inhibitors (Complete EDTA-free Protease Inhibitor Cocktail, Roche). The candidate inhibitor was added at indicated concentrations; 2,4-pyridinedicarboxylic acid (2,4-PDCA) (Sigma Aldrich) was at 5 µM. Reactions were incubated for 3 h at 37°C, stopped by 2× Laemmli loading buffer addition and directly loaded on gels for western blot analysis. Histone levels were quantified by coomassie blue stained H1 histones. Powder samples and DMSO stock solutions of 3195 were stored at −25°C

### Extraction of histones from HeLa cells

Histones were extracted from HeLa cells following the standard acid extraction procedure [34] .

### Western Blot Analysis

Yeast extracts for western blot analysis were prepared using standard glass bead disruption into a buffer A (50 mM Tris HCl at pH 7.5, 2 M Sucrose, 5 mM MgCl_2_, 1 mM DTT, Complete protease inhibitor cocktail), 45 min at 4°C. Lysed cells were centrifuged at 3100 rpm for 15 min at 4°C and pellets were resuspended in buffer B (20 mM HEPES pH 7.5, 1.5 mM MgCl_2_, 0.5 M NaCl, 0.2 mM EDTA, 20% Glycerol, 1% Triton X-100, 1 mM DTT, Complete Protease Inhibitor Cocktail (Roche).Yeast and HeLa acid extracts were separated on 15% SDS-PAGE polyacrilamide gels and transferred on nitrocellulose membranes (Whatman) by TransBlot method (Bio-Rad) in 25 mM Tris, 192 mM Glycine, 20% Methanol, 1 h at100 V at 4°C.

To characterize HIF-1 expression total cell lysates were obtained using RIPA lysis buffer (150 mM NaCl, 50 mM Tris pH8.0, 0.5% sodium deoxycholate, 0.1% SDS, 1% Nonidet P-40) containing Complete Protease Inhibitor Cocktail (Roche) and were separated on 7.5% SDS-PAGE polyacrilamide gels. Membranes were sequentially hybridized with the following antibodies: H3 (Active Motif, rabbit polyclonal 1∶1000); anti-H3K4me3 (Cell Signaling, rabbit polyclonal, 1∶1000); anti-tri-methyl-H3K36 (H3K36me3) (Active Motif, rabbit monoclonal, 1∶1000); anti-tri-methyl-H3K9 (H3K9me3) (Cell Signaling, rabbit monoclonal, 1∶1000); anti-tri-methyl-H3K27 (H3K27me3) (Cell Signaling, rabbit monoclonal, 1∶1000). HIF-1 antibody was from BD Transduction Laboratories (1∶500). 1∶25000 HRP-conjugated anti-rabbit and anti-mouse (Abcam) were used as secondary antibodies. Chemiluminescence signals intensity ratios were quantified by chemoluminescence imaging with the ChemiDoc™ XRS (Bio-Rad).

### Growth and treatment of HeLa cells

HeLa cells were grown in Dulbecco modified Eagle's minimal essential medium (DMEM) containing 10% of fetal bovin serum (FBS), 1% penicillin/streptomicin and 1% L-glutamine. HeLa cells were plated at a density of 22000 cells/cm^2^ in 35 mm diameter plates and grown at 37°C in 5% CO_2_. After 24 h compound 3195, DMSO or desferrioxamine (DFOM) were added at the indicated concentrations in the growth medium and cells were grown for an additional 24 h._._

### Flow-cytometry

Flow-cytometry analysis of DNA content was carried out using an EPICS xl flow-cytometer (Beckman-Coulter). Control and inhibitor treated HeLa cells were recovered by trypsinization, cellular pellets were washed with 5 ml of PBS and finally resuspended in PBS containing 0.1% Triton and 40 µg/ml propidium iodide (Sigma P-4170). After 20 min incubation at 37°C the samples were analyzed. The DNA content of yeast cells was determined by analyzing propidium iodide-associated fluorescence (FL3 parameter) on a linear amplification scale for cell-cycle distribution and on a logarithmic amplification scale for putative apoptotic (hypo-diploid) cell determination. 10000 events were acquired for each sample. Acquired data were analyzed using the WinMDI software by Joe Trotter, available at http://facs.scripps.edu.

### Cytoxicity assay

The cytoxicity assay was performed with Cell Counting Kit-8 (Sigma-Aldrich) according to the manifacturer's instructions.

## Results

### A model system to screen for H3K4 specific HDM inhibitors

**Figure 1:**
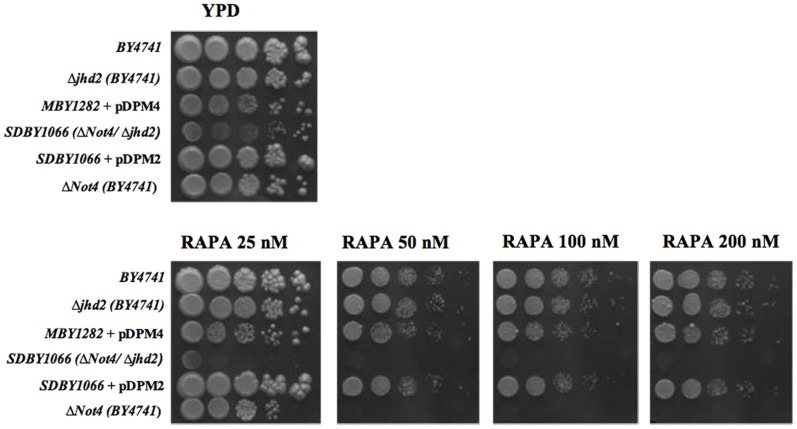
The *S.cerevisiae Δnot4/Δjhd2* strain is hypersensitive to rapamycin. Sensitivity of the indicated strains to rapamycin. Equal number of cells were plated in the absence or presence of the indicated rapamycin concentrations. Strain genotypes are listed in Table 1.

The yeast *S.cerevisiae* is an excellent system to screen for enzymes inhibitors. It is often possible to characterize strains whose genetic background requires an enzymatic activity in order to efficiently grow in particular conditions, a situation which allows massive screenings of libraries of potentially effective substances. This strategy seemed really ideal in searching for inhibitors of histone demethylases specific for H3K4 that, although existing in multiple forms in mammalians, have a unique orthologue in *S.cerevisiae.* This orthologue called Jhd2 shares a high degree of homology to mammalian JHDMs, mainly within the catalytic domain (JmjC-domain) (see Fig. S1). Deletion of *JHD2* in *S.cerevisiae* neither slows down growth rate nor provokes any evident phenotype [35]. We discovered that deletion of *JHD2*, in conjunction with deletion of *NOT4*, a gene coding for a protein involved *JHD2* post-transcriptional regulation [36], but also in many other cell regulatory processes such as RNA processing, proteolysis and transcription elongation [37], shows an evident conditional phenotype. Indeed, Fig. 1 shows that while a strain deleted in *NOT4* is moderately sensitive to rapamycin (it does not grow at concentrations higher than 25 nM), a strain deleted in *JHD2* can efficiently grow up to 200 nM rapamycin. On the other end, the double deletion strain *Δjhd2-Δnot4 SDBY1066* is not able to grow even at 25 nM rapamycin. Strikingly, the same strain transformed with the pDPM2 plasmid expressing Jhd2 (*SDBY1066*-pDPM2) grows efficiently not only at 25 nM rapamycin but even up to 200 nM. A possible explanation for this suppressive effect is that the episomal copy of Jhd2, even if under control of its own promoter, is transcribed more efficiently than the chromosomal copy in *BY4741* (around ten fold, Fig. S2). Fig. S3 shows that the conditional phenotype is evident also in liquid cultures where Jhd2 expression is required for the *SDBY1066* strain to grow in YPD medium containing 75 nM rapamycin. The *SDBY1066*-pDPM2 strain, which absolutely requires Jhd2 activity to efficiently grow in rapamycin, is therefore suitable to be exploited as selective system in searching for effective Jhd2 inhibitors.

### A Jhd2 specific inhibitor

To the best of knowledge, JmjC inhibitors act mainly by mimicking the α-KG cofactor [29]–[31]. With the aim of discovering new scaffold suitable for Jhd2 Jmjc domain inhibition, we followed a computer aided drug design approach to filter an in-house compounds library (about 6000 molecules), which led to the selection of 45 structurally unrelated derivatives, predicted to mimic the α-KG cofactor. From the available JmjC crystal structure (pdb code 2GP5, ref. [38]), we identified the main α-KG interactions in its binding site, namely the chelation of the Fe(II) atom by the α-ketocarboxylic group and a ionic interaction between the second carboxylic group and a conserved positively charged residue, and some H-bonds.

The analyses of the stuctures of known inhibitors, highlighted that they show three structural features: (i) a bidentate chelating moiety that stabilizes the iron atom, (ii) a carboxylic moiety that binds to a positively charged residue, shared with α-KG, and (iii) a methylated-lysine mimicking residue [31]. These observations led us to draw a pharmacophore model by means of which we filtered our training set. The selected compounds were then re-filtered by a sort of docking protocol versus both *JARID1B* and jhd2 homology-modelled structures.

Homology modelling studies were carried out only for the JmjC subunit of the *JARID1B* and Jhd2, because of its catalytic role. The highly conserved sequences for the JmjC domains [39] allowed to build a robust model for predicted 3D structures. Docking experiments carried out with Autodock and Plants, were able to reproduce the binding mode of α-KG and its closest analogue N-Oxalylglycine (NOG). It is worthy to note, that either Plants or Autodock failed to score the right binding pose as the best or the largest cluster, as a consequence of the force field parameterization for bonds with metal ions. Thus, we carried out superimposition studies into the JmjC catalytic domain of the α-KG bidentate chelating group with the compounds from the training set. After the alignment, the derivatives that fit the binding space well by forming of H-bond and/or hydrophobic interactions, were selected for the biological evaluation. As the residues involved in the binding of the iron and α-KG cofactor are highly conserved among JmjC domains, interactions with these residues affect inhibitory activity and decrease selectivity. Due to this reason, in our selection process we favored compounds that fitted the pharmacophore features and made contacts with residues locate at the edge of the binding site.

In order to select from the compound library molecules able to affect the *SDBY1066*-pDPM2 strain growth we inoculated exponentially growing cells in microtiter plate wells containing 200 µl of YPD plus 50 nM rapamycin. All compounds of the library were added at 5 or 15 µM. Only 3 out of 45 compounds tested showed significant (at least 75%) growth inhibition relative to the untreated sample growing in rapamycin. None of the three compounds inhibited the wild type strain growth in rapamycin. Growth in the microtiter wells is not optimal, due to the absence of efficient shaking. We therefore tested the three candidate inhibitors on larger (50 ml) cultures grown with vigorous shaking. In these conditions, only one of the inhibitors (compound 3195) showed a reproducible effect.

**Figure 2:**
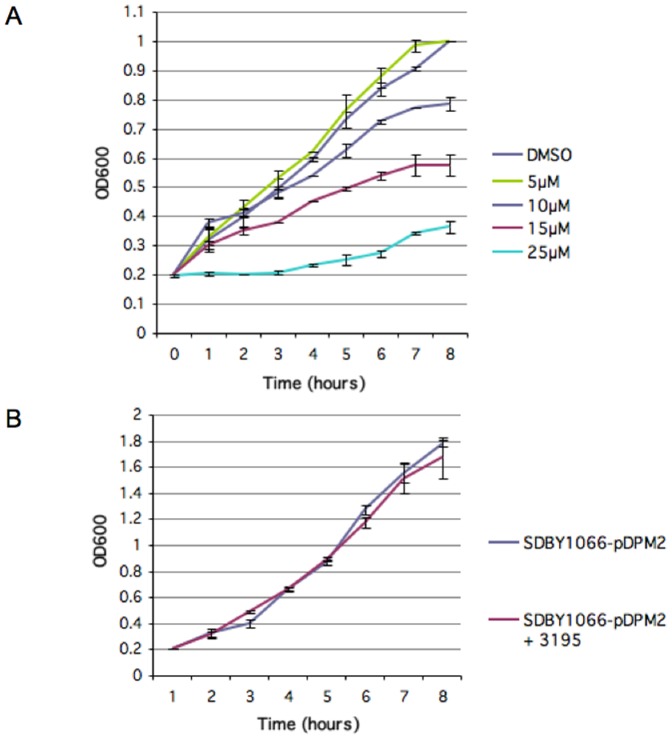
Compound 3195 inhibits growth of the pDPM2-trasformed *SDBY1066* strain in the presence of 50 nM rapamycin. (**A**) Cultures of the pDPM2-trasformed *SDBY1066* strain were diluted to 0.2 OD^600^/ml in YPD containing 50 nM rapamycin and 1.5% DMSO or increasing concentrations of 3195, as indicated in the legend, and incubated at 30°C. Data are the average of three independent growth curves, variability is reported. (**B**) Cultures of the pDPM2-trasformed *SDBY1066* strain were diluted to 0.2 OD^600^/ml in YPD containing 1.5% DMSO or 15 µM 3195 and incubated at 30°C. Data are the average of three independent growth curves, variability is reported.

Table S1 shows the effects of compound 3195 in a typical experiment on microtiter plate. The compound shows a clear growth inhibition at 24 h both at 5 and 15 µM, while at 48 h only at higher concentration shows inhibition. Fig. 2A shows the effects of different concentrations of 3195 on the *SDBY1066*-pDPM2 strain growing in 50 ml of YPD in the presence of 50 nM rapamycin with vigorous shaking: there is an increasing growth inhibition in function of inhibitor concentration, starting at 10 µM. Control experiments showed that the inhibitor has no effect in the absence of rapamycin (Fig. 2B) or on the wild type strain growing in YPD with or without rapamycin (not shown).

**Figure 3:**
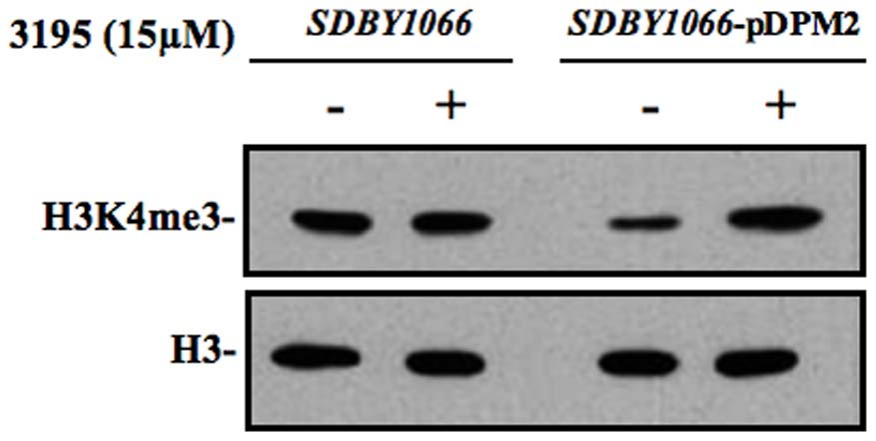
Compound 3195 increases H3K4 tri-methylation *in vivo*. NEs prepared from *SDBY1066* strain cells (left) or pDPM2-trasformed *SDBY1066* strain cells (right) incubated 4 h in the presence of 15 µM 3195 (+) or 1.5% DMSO (−) were analyzed by western blot. The same filter was hybridized with antibodies against H3K4me3 and H3 (loading control).

**Figure 4:**
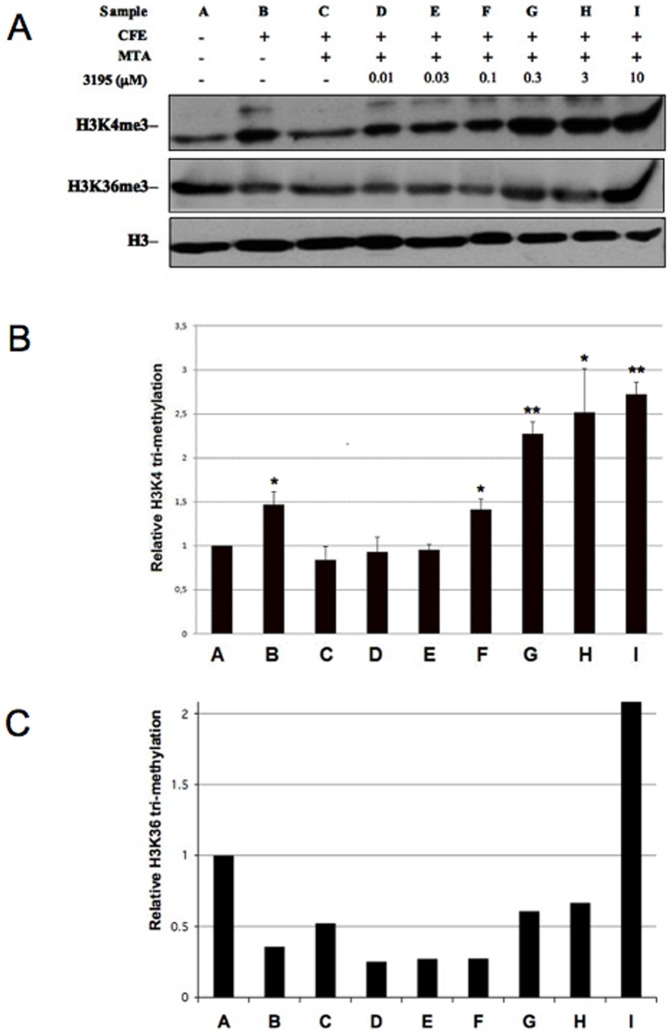
Compound 3195 inhibits H3K4 demethylase activity in *S.cerevisiae* CFE. (**A**) 5 µg of purified calf thymus histones were incubated 5 h without (lane A) or with 12 µg CFE (lanes B–I) prepared from the *S.cerevisiae* pDPM2-trasformed *SDBY1066* strain, in the presence of 1 mM α-KG, 0.1 mM Fe_2_SO_4_ and 2 mM Ascorbate. MTA 100 µM and compound 3195 were added as indicated. After incubation the samples were run in a 15% SDS PAGE gel for western blot analysis. The same filter was hybridized in succession with antibodies against H3K4me3, H3K36me3 and H3. (**B**) Quantitation of the relative fold change in H3K4me3 upon incubation with the indicated concentrations of 3195. Data are normalized to the untreated control and are the average of three independent experiments. Standard deviation is reported. Asterisks indicate significant differences relative to the reference sample (no inhibitor, sample C) according to Student t-test results (* = P<0.05; ** = P<0.01). (**C**) Quantitation of the relative fold change in H3K36me3 upon incubation with the indicated concentrations of 3195. Data (from panel A) are normalized to the untreated control.

We verified the effects of the inhibitor on H3K4 tri-methylation *in vivo*. To this purpose we prepared NEs from the *SDBY1066* strain transformed or not transformed with pDPM2 after 4 h of growth in presence of the inhibitor (or of 1.5% DMSO) and analyzed H3K4 tri-methylation level by western blot. As evident in Fig. 3, the amount of H3K4me3 detected in the double deletion strain is substantially lowered in the same strain transformed with pDPM2, an expected consequence of the ectopic expression of Jhd2 demethylase activity. Strikingly, addition of the inhibitor increases the level of H3K4me3 only in the transformed strain, while it does not have any effect on the untransformed *SDBY1066* strain. Although this experiment strongly indicates Jhd2 demethylase activity as the target of the inhibitor, it is still possible that the compound increases H3K4 methylation by an unknown and indirect way. To rule out this hypothesis we prepared CFE from the *SDBY1066*-pDPM2 strain and tested them for H3K4 methylase/demethylase activity *in vitro*. We complemented the extract with purified calf thymus histones partially tri-methylated in H3K4 and with a buffer containing Fe^2+^ and α-KG cofactors and establishing pH and ionic conditions suitable for the demethylase reaction (see Experimental Procedures for details). Fig. S4 shows that the extract itself has a prevalent H3K4 methylase activity which could mask Jhd2 demethylase effect (as expected since the Set1 histone methylase is known to be very active on *S.cerevisiae* cells). For this reason, in the CFE experiments, the HDM inhibitor assay was carried out in the presence of the Set1 histone methylase inhibitor MTA [40] which depresses the methylase effect (compare lanes B and C in Fig. 4). We then tested increasing concentrations of our candidate Jhd2 inhibitor and observed a concentration-dependent increase of H3K4me3 (Fig. 4, lanes D–I). The minimal concentration which gave a significant effect was 0.1 µM, while at 1 µM the increase seems to reach a plateau. We also tested if this effect was specific for Jhd2. We provide three evidences that this is the case:

- The same western blot, shown in Fig. 4A, was hybridized with antibodies against H3K36me3. CFE addition decreases H3K36me3 in most of the samples as compared with the untreated control, showing that a dominant demethylase activity specific for this modification is present in the extract. On the other hand, an evident increase in H3K36me3 is observed only at the highest concentration of 3195 (compare histograms reported in B and C). Thus although the H3K36 demethylases Jhd1 and Rph1 have catalytic sites similar to that one of Jhd2 and share the same chemical mechanism, our inhibitor seems more specific for Jhd2.
- We tested inhibition of H3K4 demethylation in a CFE obtained from the untrasformed *SDBY1066* strain (Fig. S5). In this case no significant effect was observed, consistent with the absence of Jhd2 activity.
- Since it was still possible that 3195 could induce an increase of H3K4 tri-methylation by interfering with the MTA inhibitory action, we tested NEs derived from a strain carrying a *SET1* gene deletion and therefore devoid of H3K4 methylase activity (Fig. S6A). Since the demethylase activity of KG obtained from this strain was very weak (not shown), we transformed it with the PDM4 plasmid carrying the *JHD2* gene under the control of a strong constitutive promoter. The NEs obtained by the transformed strain showed a significant demethylase activity which was inhibited by 3195 at concentrations ≧3 µM (Fig. S6B and C). This ruled out that the effect of 3195 could be mediated by stimulation of Set1 methylation activity.

### Testing the 3195 compound on mammalian HDMs

**Figure 5:**
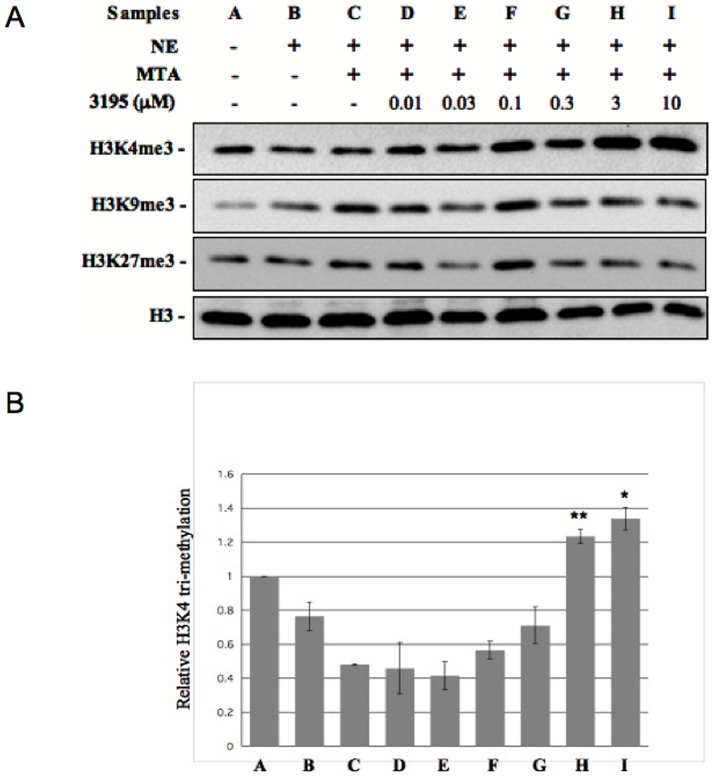
Compound 3195 inhibits H3K4 demethylase activity in HeLa NEs. (**A**) 5 µg of purified calf thymus histones were incubated 3 h with 12 µg of NE prepared from HeLa cells, in the presence of 1 mM α-KG, 0.1 mM Fe_2_SO_4_ and 2 mM Ascorbate. MTA and compound 3195 were added as indicated. After incubation, the samples were run in a 15% SDS PAGE gel for western blot analysis. The filter was hybridized in succession with antibodies against H3K4me3, H3K9me3, H3K27me3 and H3. (**B**) Quantitation of the relative fold change in H3K4me3 upon incubation with the indicated concentrations of 3195. Data are normalized to the untreated control and are the average of three independent experiments. Standard deviation is reported. Asterisks indicate significant differences relative to the reference (no inhibitor, sample C), according to Student t-test results (* = P<0.05; ** = P<0.01).

**Figure 6:**
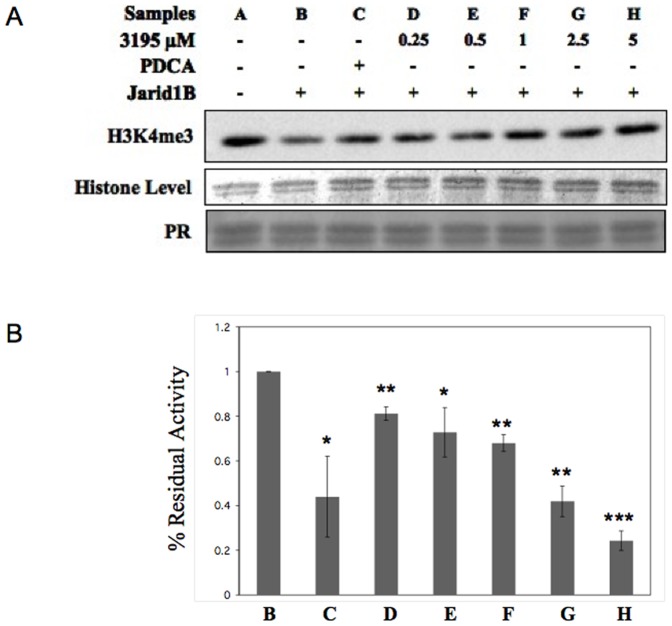
Compound 3195 inhibits *JARID1B* H3K4 demethylase activity. (**A**) 5 µg of purified calf thymus histones were incubated with 240 ng of purified *JARID1B*, in the presence of 1 mM α-KG, 0.1 mM (NH_4_)_2_Fe(SO_4_)_2_ and 2 mM Ascorbate. 2,4-PDCA (PDCA), 3195 compound (3195) or DMSO were added as indicated. After 3 h of incubation samples were run on a 15% SDS PAGE gel for western blot analysis. The filter was hybridized with antibodies against H3K4me3. Histone levels are based on coomassie blue staining. The Ponceau Red stained filter is also shown (PR). (**B**) Quantitation of the relative H3K4me3 demethylation upon incubation of *JARID1B* and the indicated concentrations of 3195. Data are adjusted to histone levels and normalized to the DMSO control, arbitrarily set as 1. Data are the average of three independent experiments. Standard deviation is indicated.

In mammalian cells at least four JHDMs exist which specifically demethylate H3K4: *JARID1A*, *1B*, *1C* and *1D* [41]. . Since the catalytic site of all the mammalian JHDMs is very conserved (Fig. S1) we expected the 3195 compound to be able to inhibit all these enzymes at least *in vitro*. Initially we tested an HeLa NE which should contain all the HDM activities. The experimental conditions were similar to that of the *S.cerevisiae* CFE assay described above. Also in the case of HeLa NE, we added MTA to repress eventual H3K4 histone methylase activities present in the extract. From the results which are presented in Fig. 5 it is evident that:

- The incubation of histones in the presence of the HeLa NE determines a slight but appreciable decrease of H3K4 tri-methylation level suggesting that, contrary to the yeast situation, in HeLa NE histone methylase activity is not prevalent on the HDMs activity (Fig. 5B).
- As expected, MTA enhances the H3K4 demethylation effect of the HeLa NE (Fig. 5A and B, sample C)
- 3195 addition at a concentration of 3 or 10 µM leads to a significant increase in H3K4 tri-methylation (2.7 and 2.9-fold average increase, as compared with CFE-MTA treated sample, respectively). We also tested possible effects on H3K9 and H3K27 tri-methylation. No clear effects were observed (Fig. 5A). In order to rule out an indirect effect of the compound, we tested the inhibitory action of 3195 on the *in vitro* H3K4 demethylase activity of purified recombinant *JARID1B* and *1D*. Fig. 6 shows that *JARID1B* is around 50% inhibited by 2,4-PDCA at a concentration of 5 µM as previously described [42]. The 3195 compound appears even more effective in inhibiting the enzyme (IC50 between 1 and 2.5 µg). Moreover it inhibits *in vitro JARID1D* activity with an IC50 of about 2 µg (Fig. S7) while it does not effectively inhibit KDM6B in the same range of concentrations (Fig. S7). Taken together, our results indicate that compound 3195 is an effective and specific inhibitor of some or all of the H3K4-specific *JARID* HDMs *in vitro*.

### Testing the effects of 3195 on HeLa cells in vivo

**Figure 7:**
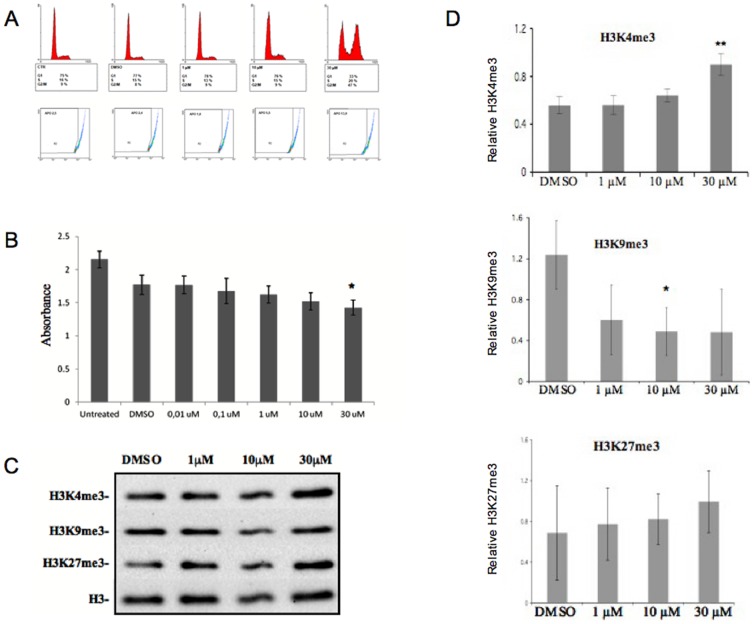
*In vivo* effects of the 3195 compound on HeLa cells. (**A**) Flow-cytometry analysis of HeLa cell of DNA content after 24 h incubation with different concentrations of 3195. Flow-cytometry analysis was carried out as described in the experimental procedure section. Fluorescence and inferred cell cycle distribution (top panel) and putative percent of apoptotic cells (bottom panel) are reported. (**B**) Cytotoxicity assay: the histogram reports the absorbance measured at 450 nm which is proportional to the number of alive cells and represent the average of four independent experiments. Standard deviation is reported. Asterisk indicates significant difference between inhibitor treated cells and DMSO control cells, according to Student's t test results (* = P<0.02).(**C**) Western blot analysis shows H3K4me3, H3K9me3, H3K27me3 levels and total H3 level in the HeLa cell acid protein fractions after 24 h of treatment with the indicated concentrations of compound 3195 or with DMSO. (**D**) Quantitation of data obtained as in panel C. Data refer to 3 (H3K9me3 and H3K27me3) or 4 (H3K4me3) independent experiments. Standard deviation is reported. Asterisks indicate where the change in tri-methylation induced by the compound 3195 is significant, according to Student t-test results (* = P<0.05; ** = P<0.01).

We tested the effects of different concentrations (1, 10 and 30 µM) of 3195 added to HeLa cells directly in the growth medium. At the concentration of 30 µM the compound strongly affected cell cycle dynamics as determined by flow-cytometry analysis (Fig. 7A). In particular, a strong increase of cells in G2/M (47% as compared with 9% of the untreated or DMSO treated controls) was observed after 24 h of treatment with 3195. This increase corresponds to a net decrease of G0–G1 cells (33 vs 75–77%) indicating that the cells are preferentially blocked after completion of DNA synthesis. The flow-cytometry analysis showed also a 10% increase of potentially apoptotic cells. This mild cytoxic effect of 3195 is confirmed by the cytotoxicity assay (Fig. 7B) which showed a presumptive induced mortality around 25%, as compared with DMSO treated samples. In order to correlate these effects with the inhibitory action on demethylase activity, we tested the effect of the same 3195 concentrations on H3K4, K9 and K27 tri-methylation by monitoring the steady-state levels of modification *in vivo*. Fig. 7C shows that the addition of the highest 3195 concentration (30 µM) leads to a strong increase in H3K4 and H3K27 tri-methylation, while H3K9 tri-methylation levels seem to remain constant or even to decrease. The increase in H3K4me3 is statistically significant while the increase in H3K27me3 and the decrease in H3K9me3 appear less reproducible (Fig. 7D).

Finally, since it was shown that a-KG mimics may inhibit human Hypoxia-Inducible-Factor (HIF) hydroxylases PHD1 and PHD2 [43] causing HIF accumulation and consequent cancer cell protection, we tested 3195 for induction of HIF1α accumulation in HeLa cells. Fig. S8 shows that, while the iron chelator desferrioxamine (DFOM), used as positive control, is very effective in inducing HIF1α accumulation as decribed [44], 3195 does not show any effect even at 30 µM, ruling out a significant inhibition of the PHD hydroxylases *in vivo*.

## Discussion

JmjC (Jumonji C) domain-containing proteins are known to be an extensive family of Fe(II)/2-oxoglutarate-dependent oxygenase involved in all eukaryotes in many different biological tasks such as protein stabilization, hypoxia sensing and fatty acid metabolism [45]. Recently they have been involved in DNA and histone demethylation, and their role is further expanding [46]. Although it is relatively easy to screen for JHDMs inhibition *in vitro* [27], predicting *in vivo* efficacy on the base of *in vitro* inhibitory activity is not straightforward [29]. An interesting *in vivo* screening system for small molecules capable to interfere with transcription repressive epigenetic modifications was recently described [47]. The system proved to be able to identify a new molecule which inhibits Jumonji HDMs but with low selectivity between the various class.

We describe here an high-throughput *in vivo* screening system which has important features that makes it ideal for the selection of H3K4-specific inhibitors of JHDMs potentially able to enter eukaryotic cells and to act on their putative targets without affecting other HDMs or other important 2-oxoglutarate oxygenases. First, it is based on the yeast *S.cerevisiae* which has a unique and not-essential JHDM specific for H3K4 whose catalytic site is highly homologous to mammalian JHDMs. Second, it makes use of a double deletion strain (*Δnot4/Δjhd2*) which relies on over-expression from an episomal copy of Jhd2 (*SDBY1066*-pDPM2) in order to efficiently grow in the presence of rapamycin, a feature which is correlated to an enhanced biological sensitivity to inhibitors. Third, being the demethylase over-expressed and its activity less stringently regulated and more comparable with the strong Set1 histone methylase activity, its inhibition produces an increase of H3K4me3 much more evident than the 20–30% increase which is generally observed in *jhd2*-deleted strains [16], [35]. Fourth and most importantly, secondary effects on other Fe(II)/2-oxoglutarate-dependent oxygenases can be easily identified by testing the cytostatic effects of candidate compounds in the absence of rapamycin or on two different kinds of control strains: wild type strains or strains devoid of Jhd2 activity.

In order to test the potential of the system in identifying selective HDM inhibitors, we screened a library of 45 putative competitive inhibitors of α-KG selected by means of a computational approach. This class of compounds has previously been shown to be very effective *in vitro* and also to have the potential to discriminate among the various groups of Jumonji HDMs and between them and other important hydroxylases. On the other end, it is not straightforward to predict the most *in vivo* effective and selective compounds based on their structures or on *in vitro* results [43]. A considerable effort is currently being made to identify α-KG mimics which in a certain range of concentration can inhibit a specific c lysine modification without significantly affecting others lysines [43]. Moreover, the potential use of this class of compounds in cancer therapy is seriously limited by their eventual effect in inhibiting human Hypoxia-Inducible-Factor (HIF) PHD hydroxylases [43], causing HIF accumulation and consequent cancer cell protection. For this reasons this class of inhibitors constituted an ideal challenge for our screening system.

**Figure 8:**
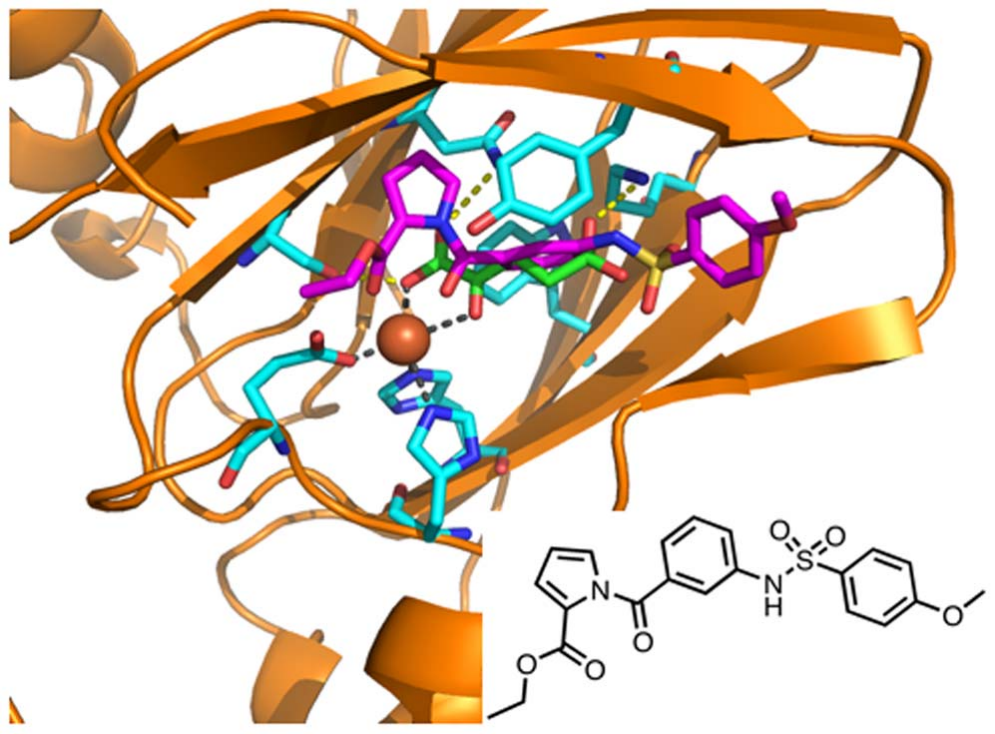
Homology modelled structure of Jhd2 JmjC. Binding site residues are reported as cyan stick. α-KG co-crystallized (in green) and docked (in red) binding poses. 3195 chemical structure (insert) and proposed binding mode (in magenta) are shown.

We found a compound (see Fig. 8) which at a concentration of 15 µM inhibited the growth of the *SDBY1066*-pDPM2 strain over-expressing Jhd2 in rapamycin, while it did not show any effect on the same strain in the absence of rapamycin or on the wild type strain with or without rapamycin. This compound effectively inhibited the H3K4 demethylase activity at the same concentration *in vivo*, while it was active at concentrations as low as 300 nM on H3K4 demethylase activity assayed *in vitro* on *S.cerevisiae* CFE. Moreover, the same compound shows a certain degree of specificity for Jhd2 since *in vitro* it has an effect on H3K36 tri-methylation only at a 100-fold higher concentration. Compound 3195 is also active on HeLa cells NE at 3 and 10 µM concentration. At the same concentrations we did not observe any increase in H3K9me3 and H3K27me3. These results suggest that, at least at the tested range of concentrations, the compound is specific for H3K4. However, we can not exclude the possibility that the Fe^2+^- and α-KG-dependent JHDMs, which demethylate these H3 lysine residues, are not active enough in the HeLa NE to show a clear effect in our assay. We measured inhibition on the purified *JARID1B* and *1D* activity and found for both of them an LC_50_ around 2 µg (in excess of α-KG), which is in the lower range of the best HDM inhibitors previously described [27]–[31], [43]. On the other end 3195 did not show a consistent inhibition *in vitro* on H3K27 demetylation by KDM6B (Fig. S6). We tested the effects of compound 3195 on HeLa cells *in vivo*. We observed a strong cytostatic effect at 30 µM, concentration at which around 47% of the cells remained blocked in G2/M (Fig. 7). At the same concentration, compound 3195 induced a mild cytoxicity, and a moderate apoptogenic effect. When we tested the induced variation in global histone H3 tri-methylation level in treated HeLa cells, we found a significant and reproducible increase for H3K4 but not for H3K9 which instead showed a tendency to decrease. An interesting possibility is that this decrease is caused by cross-talk between H3K4 and H3K9 methylases which has been recently characterized [48], but this still remains to be proven. H3K27 tri-methylation level seems also to increase but in a less reproducible way as compared with H3K4 (Fig. 7D). It was previously observed that H3K4 tri-methylation level does not significantly change in function of the cell cycle progression [49]. It is therefore very likely that the observed increase is due to a direct effect of *JARID* HDM inhibition *in vivo* and that the block in G2/M is a consequence rather than a cause of this inhibition. On the other end, an accurate quantitative analysis of H3K27 tri-methylation through the cell cycle in HeLa cells showed that there is a consistent post-replicative increase of this modification with an activity peak in G2/M [50]. This suggests that the observed increase in H3K27me3 could be due to the induced block at this stage of the cell cycle. This hypothesis is also supported by the cell cycle-regulation of H3K27 methylase Ezh2 which has been previously shown to be activated at G2/M [51].

Finally we tested 3195 for induction of HIF1α accumulation in HeLa cells and we did not find any increase at concentrations up to 30 µM (Fig. S8).

In conclusion, among 45 potential α-KG mimics screened, we fished out the inhibitor 3195 which, similarly to the recently discovered PBIT [27], shows selectivity for H3K4 demethylation without significantly affecting other lysine demethylase and other important hydoxylase activities. A direct relationship between this inhibitory action and the observed cytostatic effect as well as 3195 in depth mechanism of action still remains to be fully elucidated. Also its potential use in investigating the biological role of H3K4 specific demethylases in normal and cancer cells remains to be tested. On the other end our selection system proved to be a very promising tool for the discovery of H3K4 specific HDM inhibitors active *in vivo*.

## Supporting Information

Figure S1 **Sequence alignment of Jmc domains from the Jhd2 and the** ***JARID1*** **family members.**
(TIF)

Figure S2 **Ectopic over-expression of** ***JHD2*** **in the pDPM2-trasformed** ***Δnot4/Δjhd2*** **strain.** Real time RT-PCR was performed on cDNA obtained from total RNA extracted from the indicated strains during exponential growth. Data represent fold change relative to *ACT1* mRNA, used as endogenous calibrator, and are the average of three independent experiments. Standard deviation is reported. Asterisk indicate statistically significant changes as compared with the untreated wild type strain *BY4742*. For the untrasformed *SDBY1066* strain no amplification was obtained.
(TIF)

Figure S3 **The** ***S.cerevisiae SDBY1066*** **strain is hypersensitive to rapamycin in liquid culture.** The indicated strains were grown at 30°C in YPD until an OD^600^ of 0.4 was reached. At this point rapamycin (75 nM) was added and growth monitored for 7 h.
(TIF)

Figure S4 **H3K4 tri-methylation activity is prevalent on** ***S.cerevisiae*** **pDPM2-trasformed** ***SDBY1066*** **strain CFE.** (**A**) 5 µg of purified calf thymus histones were incubated for 3 h with the indicated amount of CFE prepared from *S.cerevisiae* pDPM2-trasformed *SDBY1066* strain, in the presence of 10 mM α-KG, 1 mM Fe_2_SO_4_ and 2 mM Ascorbate. After incubation samples were run on a 15% SDS gel for western blot analysis. The filter was hybridized in succession with antibodies against H3K4me3 and H3. (**B**) Quantitation of the relative H3K4me3 upon incubation with CFE from *S.cerevisiae* pDPM2-trasformed *SDBY1066* strain. The intensities of H3K4me3 bands were normalized to the intensity of the corresponding H3 bands. Histograms represent the average of three independent experiments. Standard deviation is reported. Asterisks indicate where the change in H3K4me3 of the CFE treated samples, as compared with the untreated control, is significant according to Student t-test results (* = P<0.05; ** = P<0.01).
(TIF)

Figure S5 **Compound 3195 has no effect on the untrasformed** ***S.cerevisiae Δnot4/Δjhd2*** **strain (** ***SDBY1066*** **).** 5 µg of purified calf thymus histones were incubated for 3 h with the indicated amount of CFE from *S.cerevisiae SDBY1066* strain, in the presence of 10 mM α-KG, 1 mM Fe_2_SO_4_ and 2 mM Ascorbate. MTA and compound 3195 were added as indicated. After incubation samples were run on a 15% SDS gel for western blot analysis. The filter was hybridized with H3K4me3 antibody. The Ponceau Red staining (PR) is shown as loading control of calf thymus histones.
(TIF)

Figure S6 **Compound 3195 inhibits H3K4 demethylase activity in CFEs prepared from a** ***Δset1*** **strain.** Panel A: 20 µg of CFEs prepared from wild type W303 or *Δset1 YCVS3* strains, as indicated, were run on a 15% SDS gel for western blot analysis. The filter was hybridized with anti-H3K4me3 to control for the absence of H3K4me3 in the *Δset1* strain. The Ponceau Red staining (PR) is shown as loading control. Panel B: 5 µg of purified calf thymus histones were incubated for 3 h with 12 µg of CFE prepared from *S.cerevisiae Δset1 YCVS3* strain transformed with pDPM4, in the presence of 10 mM α-KG, 1 mM Fe_2_SO_4_ and 2 mM Ascorbate. Compound 3195 was added as indicated. After incubation samples were run in a 15% SDS gel for western blot analysis. The filter was hybridized with anti- H3K4me3 and anti-H3 antibodies. The Ponceau red staining (PR) is shown as loading control of calf thymus histones. Panel C: Quantitation of western blot analysis of the relative H3K4me3 demethylation upon histone incubation with *S.cerevisiae* Δset1 *YCVS3* strain CFEs and different concentrations of 3195. H3K4me3 data were normalized to the untreated control (lane A), arbitrarily set as 1, and are the average of three independent experiments performed with three different CFEs. Standard deviation is indicated. Asterisks indicate where changes in H3K4me3 of the CFE treated samples, compared to the untreated control, are significants according to Student t-test results (* = P<0.05; ** = P<0.01).
(TIF)

Figure S7 **Compound 3195 inhibits** ***JARID1D*** **H3K4 demethylase activity but does not affect KDM6B activity.** (**A**) 5 µg of purified calf thymus histones were incubated 1 h with 105 ng of purified *JARID1D*, in the presence of 1 mM α-KG, 0.1 mM (NH_4_)_2_Fe(SO_4_)_2_ and 2 mM Ascorbate. Compound 3195 or DMSO were added as indicated. After incubation samples were run on a 15% SDS page gel for western blot analysis. The fliter was sequentially hybridized with antibodies against H3K4me3 and H3. Histone levels are based on coomassie stain. (**B**) Quantitation of the relative H3K4me3 demethylation upon incubation with *JARID1D* and the indicated concentrations of 3195. Data are adjusted to histone levels and normalized to the DMSO control, arbitrarily set as 1. Data are the average of three independent experiments. Standard deviation is indicated. (**C**) 5 µg of purified calf thymus histones were incubated 3 h with 80 ng of purified *KDM6B*, in the presence of 1 mM α-KG, 0.1 mM (NH_4_)_2_Fe(SO_4_)_2_ and 2 mM Ascorbate. DMSO, compound 3195 or 2,4-PDCA (PDCA) were added as indicated. 2,4-PDCA was at 5 µM final concentration. After incubation samples were run on a 15% SDS page gel for western blot analysis. The filter was hybridized with antibodies against H3K27me3. The Ponceau red stained filter (PR) is shown as loading control of calf thymus histones.
(TIF)

Figure S8 **3195 compound does not affect HIF-1 expression in Hela cells.** Western blot analysis shows HIF-1 levels in HeLa cell lysates after 24 h of treatment with the indicated concentrations of compound 3195, DMSO or DFOM. 50 µg of total proteins were loaded on SDS-PAGE gels.
(TIF)

Table S1 **Effect of compound 3195 on pDPM2-transformed** ***SDBY1066*** **strain cells grown in rapamycin.** Cells were inoculated in 200 µl of YPD plus 50 nM rapamycin at a cell density corresponding to 0.2 OD^600^ and incubated at 30°C in the presence of compound 3195 or DMSO as indicated. OD^600^ were read at the indicated times. Data are the average of two wells inoculated with cells from independent cultures. Variability is indicated and significant reductions (≥75%) are shown in bold.
(DOC)
