# Supplementary figures and images for: An High-Throughput In Vivo Screening System to Select H3K4-Specific Histone Demethylase Inhibitors
Source: PLoS One. 2014 Jan 29;9(1):e86002. doi: 10.1371/journal.pone.0086002 (PMC3906020; doi:10.1371/journal.pone.0086002)

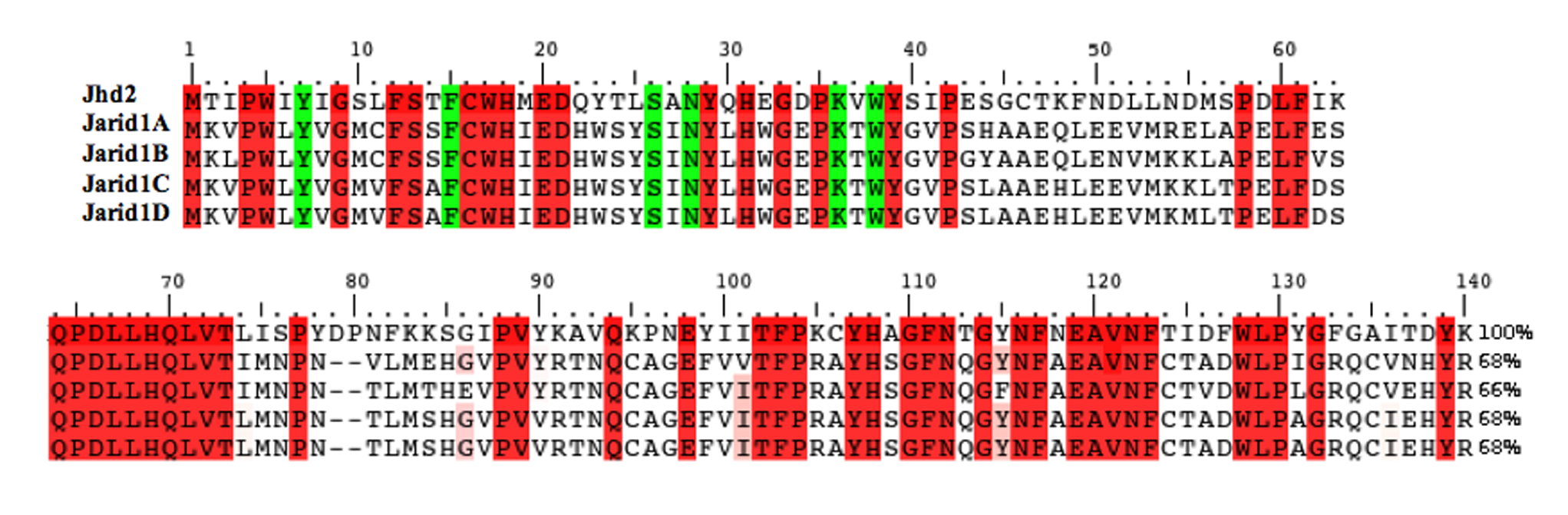

Supplement: Figure S1 — Sequence alignment of Jmc domains from the Jhd2 and the JARID1 family members. (TIF) [file pone.0086002.s001.tif]

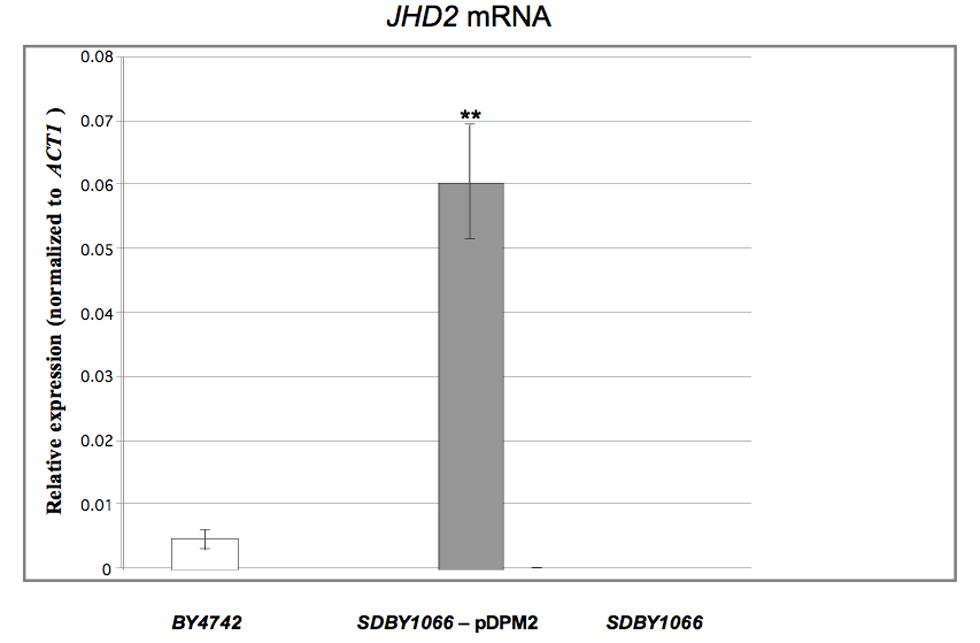

Supplement: Figure S2 — Ectopic over-expression of JHD2 in the pDPM2-trasformed Δnot4/Δjhd2 strain. Real time RT-PCR was performed on cDNA obtained from total RNA extracted from the indicated strains during exponential growth. Data represent fold change relative to ACT1 mRNA, used as endogenous calibrator, and are the average of three independent experiments. Standard deviation is reported. Asterisk indicate statistically significant changes as compared with the untreated wild type strain BY4742. For the untrasformed SDBY1066 strain no amplification was obtained. (TIF) [file pone.0086002.s002.tif]

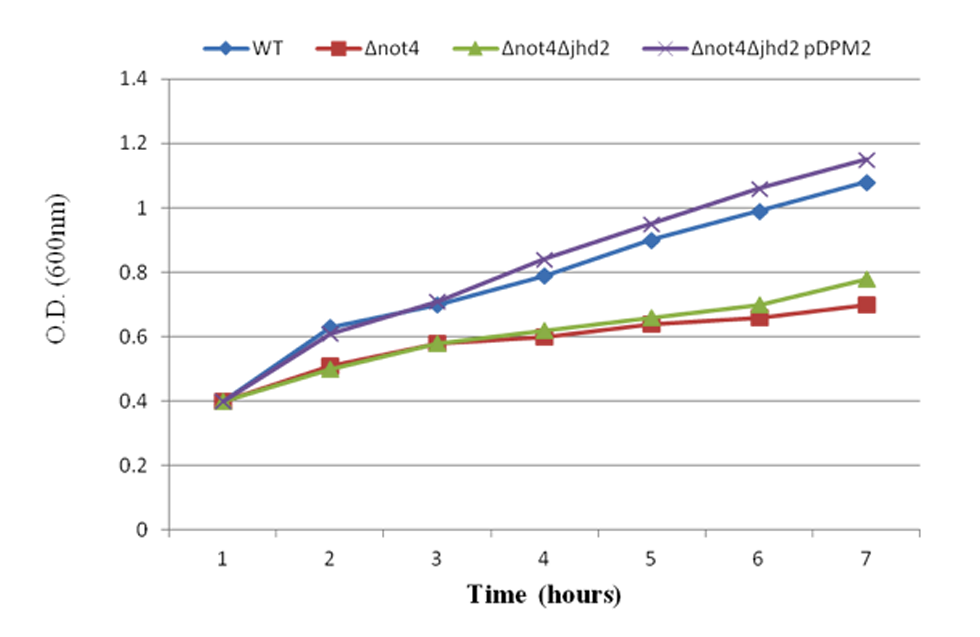

Supplement: Figure S3 — The S.cerevisiae SDBY1066 strain is hypersensitive to rapamycin in liquid culture. The indicated strains were grown at 30°C in YPD until an OD600 of 0.4 was reached. At this point rapamycin (75 nM) was added and growth monitored for 7 h. (TIF) [file pone.0086002.s003.tif]

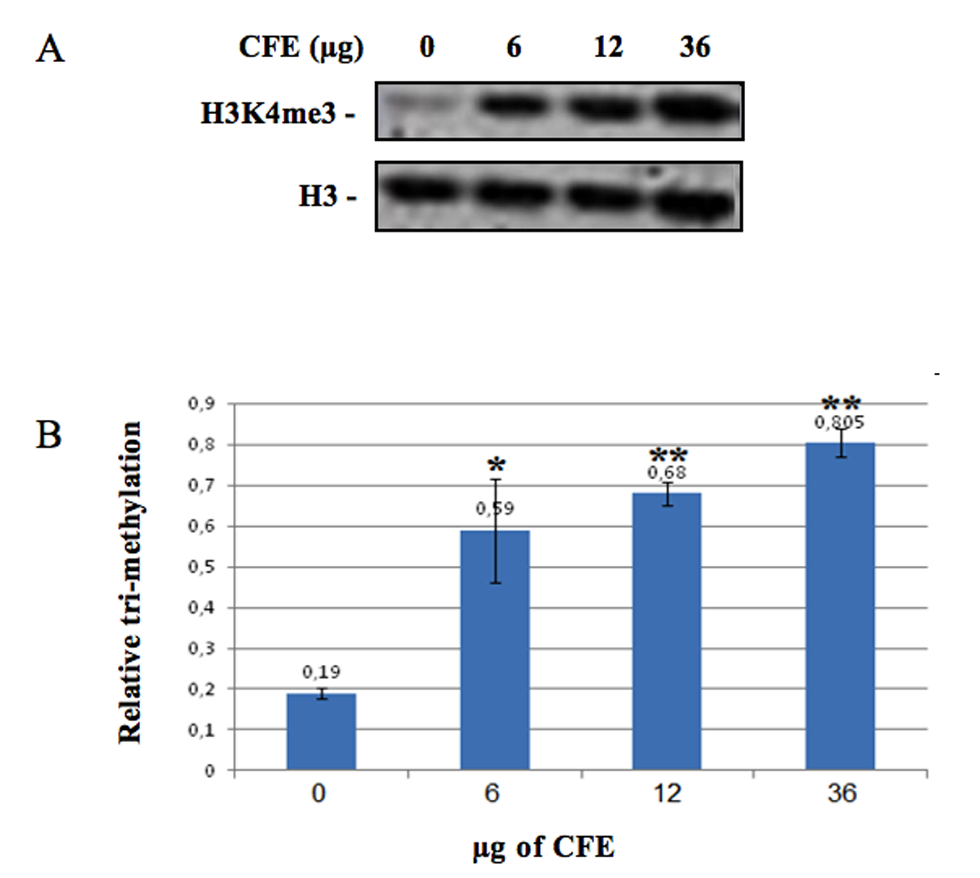

Supplement: Figure S4 — H3K4 tri-methylation activity is prevalent on S.cerevisiae pDPM2-trasformed SDBY1066 strain CFE. (A) 5 µg of purified calf thymus histones were incubated for 3 h with the indicated amount of CFE prepared from S.cerevisiae pDPM2-trasformed SDBY1066 strain, in the presence of 10 mM α-KG, 1 mM Fe2SO4 and 2 mM Ascorbate. After incubation samples were run on a 15% SDS gel for western blot analysis. The filter was hybridized in succession with antibodies against H3K4me3 and H3. (B) Quantitation of the relative H3K4me3 upon incubation with CFE from S.cerevisiae pDPM2-trasformed SDBY1066 strain. The intensities of H3K4me3 bands were normalized to the intensity of the corresponding H3 bands. Histograms represent the average of three independent experiments. Standard deviation is reported. Asterisks indicate where the change in H3K4me3 of the CFE treated samples, as compared with the untreated control, is significant according to Student t-test results (* = P<0.05; ** = P<0.01). (TIF) [file pone.0086002.s004.tif]

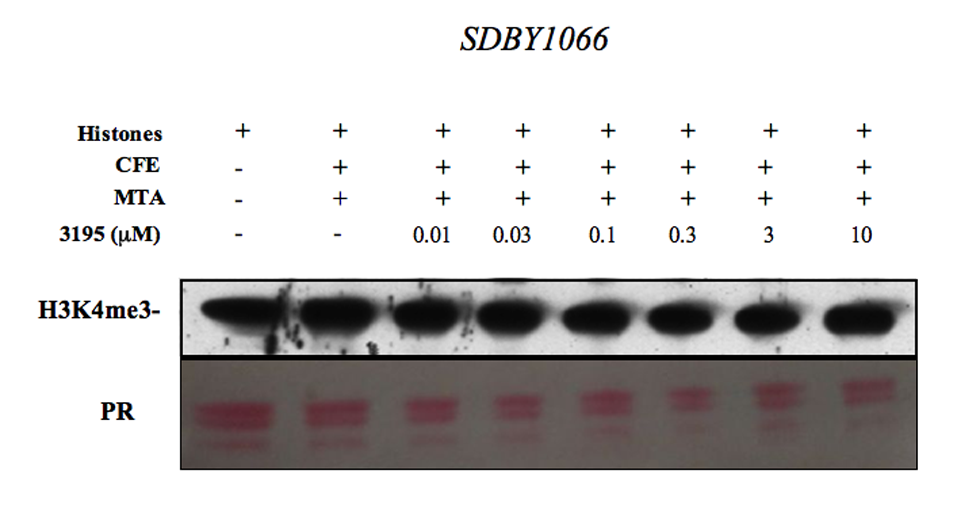

Supplement: Figure S5 — Compound 3195 has no effect on the untrasformed S.cerevisiae Δnot4/Δjhd2 strain ( SDBY1066 ). 5 µg of purified calf thymus histones were incubated for 3 h with the indicated amount of CFE from S.cerevisiae SDBY1066 strain, in the presence of 10 mM α-KG, 1 mM Fe2SO4 and 2 mM Ascorbate. MTA and compound 3195 were added as indicated. After incubation samples were run on a 15% SDS gel for western blot analysis. The filter was hybridized with H3K4me3 antibody. The Ponceau Red staining (PR) is shown as loading control of calf thymus histones. (TIF) [file pone.0086002.s005.tif]

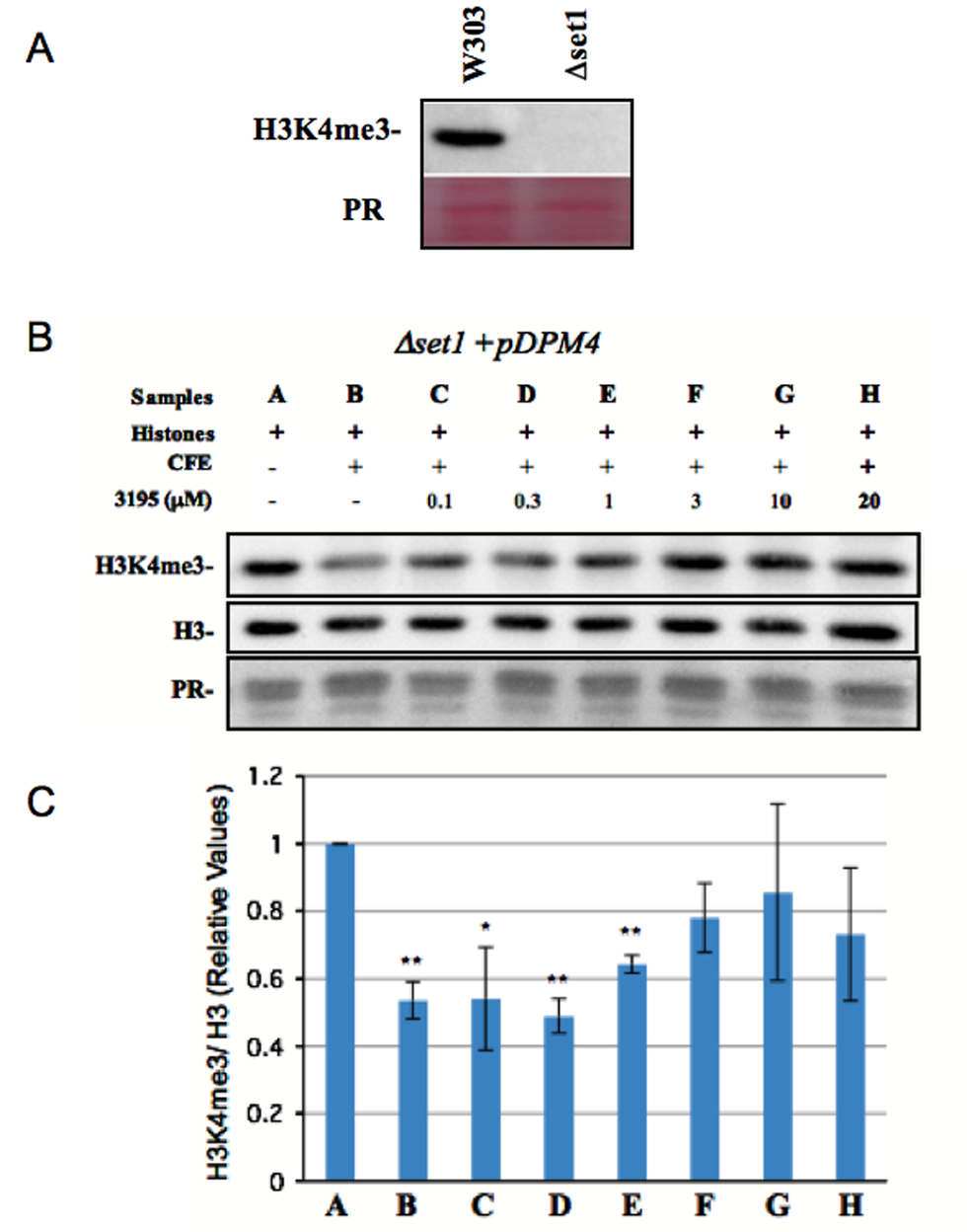

Supplement: Figure S6 — Compound 3195 inhibits H3K4 demethylase activity in CFEs prepared from a Δset1 strain. Panel A: 20 µg of CFEs prepared from wild type W303 or Δset1 YCVS3 strains, as indicated, were run on a 15% SDS gel for western blot analysis. The filter was hybridized with anti-H3K4me3 to control for the absence of H3K4me3 in the Δset1 strain. The Ponceau Red staining (PR) is shown as loading control. Panel B: 5 µg of purified calf thymus histones were incubated for 3 h with 12 µg of CFE prepared from S.cerevisiae Δset1 YCVS3 strain transformed with pDPM4, in the presence of 10 mM α-KG, 1 mM Fe2SO4 and 2 mM Ascorbate. Compound 3195 was added as indicated. After incubation samples were run in a 15% SDS gel for western blot analysis. The filter was hybridized with anti- H3K4me3 and anti-H3 antibodies. The Ponceau red staining (PR) is shown as loading control of calf thymus histones. Panel C: Quantitation of western blot analysis of the relative H3K4me3 demethylation upon histone incubation with S.cerevisiae Δset1 YCVS3 strain CFEs and different concentrations of 3195. H3K4me3 data were normalized to the untreated control (lane A), arbitrarily set as 1, and are the average of three independent experiments performed with three different CFEs. Standard deviation is indicated. Asterisks indicate where changes in H3K4me3 of the CFE treated samples, compared to the untreated control, are significants according to Student t-test results (* = P<0.05; ** = P<0.01). (TIF) [file pone.0086002.s006.tif]

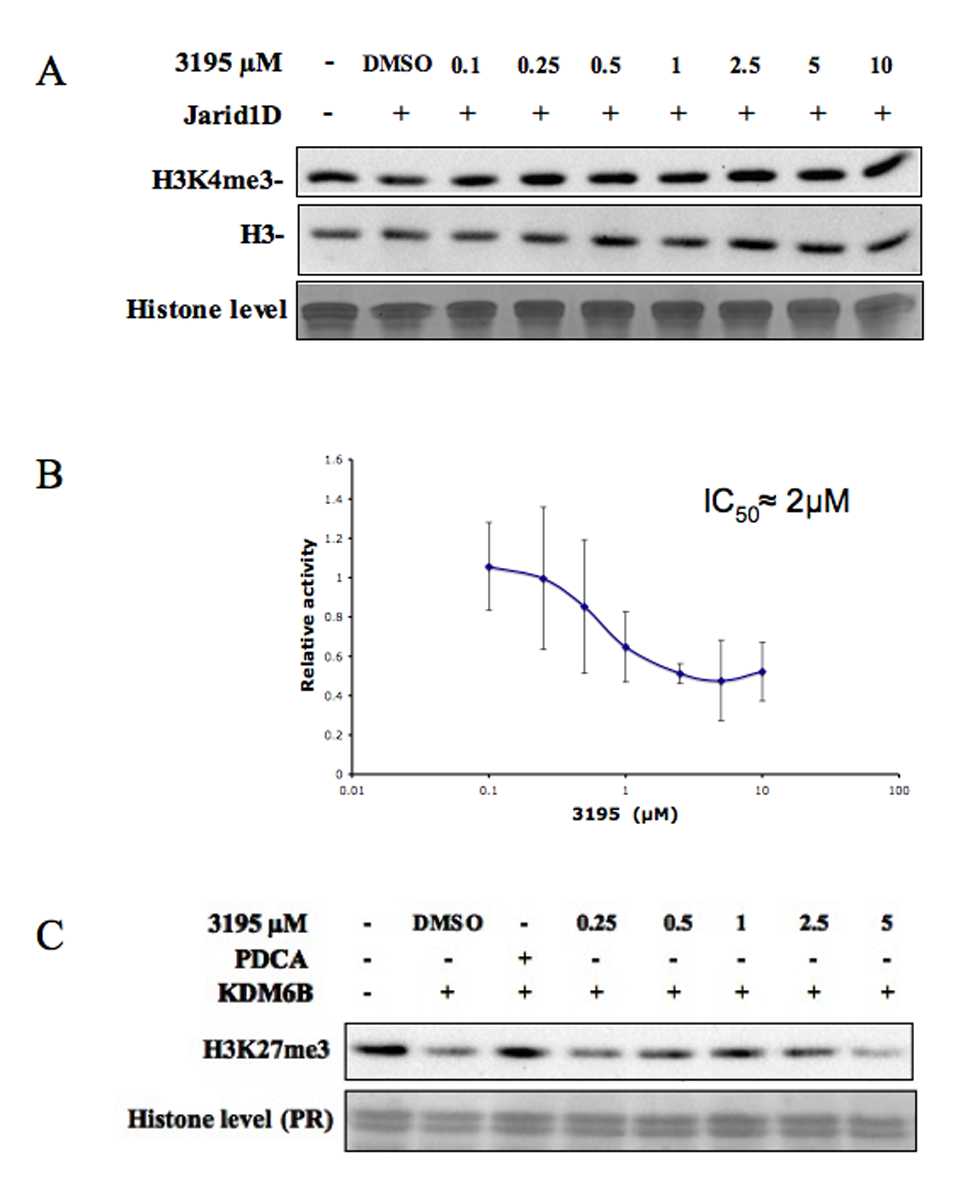

Supplement: Figure S7 — Compound 3195 inhibits JARID1D H3K4 demethylase activity but does not affect KDM6B activity. (A) 5 µg of purified calf thymus histones were incubated 1 h with 105 ng of purified JARID1D, in the presence of 1 mM α-KG, 0.1 mM (NH4)2Fe(SO4)2 and 2 mM Ascorbate. Compound 3195 or DMSO were added as indicated. After incubation samples were run on a 15% SDS page gel for western blot analysis. The fliter was sequentially hybridized with antibodies against H3K4me3 and H3. Histone levels are based on coomassie stain. (B) Quantitation of the relative H3K4me3 demethylation upon incubation with JARID1D and the indicated concentrations of 3195. Data are adjusted to histone levels and normalized to the DMSO control, arbitrarily set as 1. Data are the average of three independent experiments. Standard deviation is indicated. (C) 5 µg of purified calf thymus histones were incubated 3 h with 80 ng of purified KDM6B, in the presence of 1 mM α-KG, 0.1 mM (NH4)2Fe(SO4)2 and 2 mM Ascorbate. DMSO, compound 3195 or 2,4-PDCA (PDCA) were added as indicated. 2,4-PDCA was at 5 µM final concentration. After incubation samples were run on a 15% SDS page gel for western blot analysis. The filter was hybridized with antibodies against H3K27me3. The Ponceau red stained filter (PR) is shown as loading control of calf thymus histones. (TIF) [file pone.0086002.s007.tif]

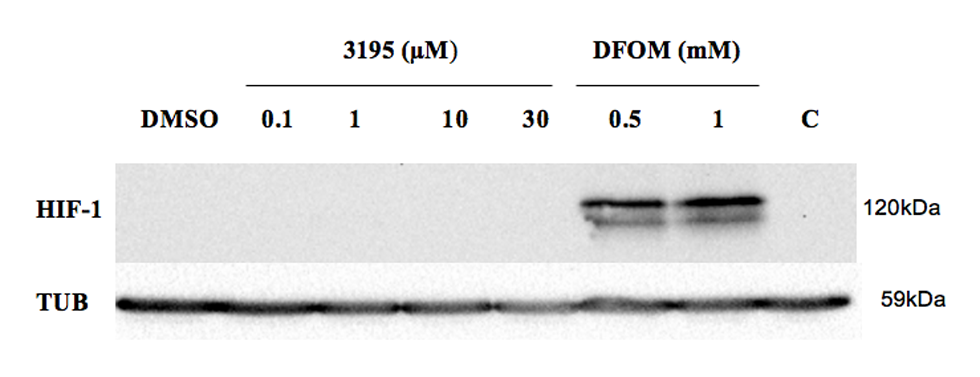

Supplement: Figure S8 — 3195 compound does not affect HIF-1 expression in Hela cells. Western blot analysis shows HIF-1 levels in HeLa cell lysates after 24 h of treatment with the indicated concentrations of compound 3195, DMSO or DFOM. 50 µg of total proteins were loaded on SDS-PAGE gels. (TIF) [file pone.0086002.s008.tif]
