# Supplementary material for: An High-Throughput In Vivo Screening System to Select H3K4-Specific Histone Demethylase Inhibitors
Source: PLoS One. 2014 Jan 29;9(1):e86002. doi: 10.1371/journal.pone.0086002 (PMC3906020; doi:10.1371/journal.pone.0086002)
Supplement: Table S1 — Effect of compound 3195 on pDPM2-transformed SDBY1066 strain cells grown in rapamycin. Cells were inoculated in 200 µl of YPD plus 50 nM rapamycin at a cell density corresponding to 0.2 OD600 and incubated at 30°C in the presence of compound 3195 or DMSO as indicated. OD600 were read at the indicated times. Data are the average of two wells inoculated with cells from independent cultures. Variability is indicated and significant reductions (≥75%) are shown in bold. (DOC) [file pone.0086002.s009.doc]

**Table S1. Effect of compound 3195 on pDPM2-transformed *SDBY1066* strain cells grown in 200** µ**l of YPD plus 50 nM rapamycin**

| Treatment | - | 1.5% DMSO | 5 µM 3195 | 15 µM 3195 |
| --- | --- | --- | --- | --- |
| OD^600^ 24 h | 1.45 ± 0.06 | 1.21 ± 0.05 | **0.21**  ± **0.02** | **0.20**  ± **0.01** |
| OD^600^ 48 h | 2.3 ± 0.12 | 2.84 ± 0.17 | 3.1 ± 0.27 | **0.23**  ± **0.02** |
